# Comparative Neurocognitive Outcomes Following Holmium Laser Enucleation and Transurethral Resection of the Prostate: A Prospective Cohort Study

**DOI:** 10.3390/medicina62050971

**Published:** 2026-05-15

**Authors:** Orkunt Özkaptan, Cengiz Çanakcı, Erdinç Dinçer, Osman Murat İpek, Mehmet Burak Doğrusever, Oğuz Türkyılmaz, Alper Coşkun, Sare Dilek Özkaptan

**Affiliations:** 1 Department of Urology, Health Sciences University, Kartal Dr. Lutfi Kirdar City Hospital, 34890, Istanbul, Turkey; cengizcanakci@hotmail.com (C.Ç.); drerdincdincer@gmail.com (E.D.); omipek@hotmail.com (O.M.İ.); burakdogrusever@hotmail.com (M.B.D.); turkylmazoguz@gmail.com (O.T.); dr.alper05@gmail.com (A.C.); 2 Department of Neuropsychology, Kartal Dr. Lutfi Kirdar City Hospital, 34890 Istanbul, Turkey; dileksare@gmail.com

**Keywords:** Benign Prostatic Hyperplasia, MoCA, HoLEP, TURP

## Abstract

*Background and Objectives*: To evaluate the impact of Holmium Laser Enucleation of the Prostate (HoLEP) versus Transurethral Resection of the Prostate (TURP) on cognitive function and psychological well-being three months post-surgery. *Materials and Methods*: This prospective observational cohort study involved 150 patients undergoing surgical treatment for BPH; 132 patients (66 HoLEP, 66 TURP) completed baseline and 3-month follow-up evaluations. The Montreal Cognitive Assessment (MoCA) served as the primary measure of cognitive function, while the Mini-Mental State Examination (MMSE) functioned as a secondary measure. The Beck Anxiety Inventory and Beck Depression Inventory were utilized to assess individuals’ mental states. We employed repeated-measures General Linear Models, adjusted for age and educational attainment, to examine temporal variations. *Results*: Baseline demographic, clinical, cognitive, and psychological characteristics were comparable among the groups. The modified analysis revealed no significant interaction between time and surgical procedure for MoCA (*p* = 0.405), indicating that both groups exhibited comparable cognitive trajectories. No significant differences were seen between the groups in the adjusted MoCA scores (*p* = 0.162). A minor, statistically insignificant temporal effect was observed (*p* = 0.058; partial η^2^ = 0.028). Educational attainment independently forecasted cognitive performance (*p* = 0.024). The MMSE demonstrated a slight temporal effect (*p* = 0.015) with no interaction of approaches. Anxiety and depressive symptoms persisted uniformly and comparably among the groups. *Conclusions*: Three months post-surgery, neither HoLEP nor TURP was associated with a notable deterioration in cognitive performance. The surgical modality did not independently influence cognitive trajectory after adjusting for demographic variables. Contemporary endoscopic BPH surgery appears to be neurocognitively safe during the medium-term postoperative period.

## 1. Introduction

Benign prostatic hyperplasia (BPH) is a common disorder impacting older males. Histological evidence indicates that approximately 80% of men over the age of 70 are affected [1]. As the prostate enlarges, it may lead to anatomical bladder outlet obstruction (BOO) and lower urinary tract symptoms (LUTS), severely affecting quality of life. For patients experiencing moderate to severe symptoms unresponsive to medical treatment, surgical intervention remains the optimal solution [2].

Transurethral resection of the prostate (TURP) has long been considered the optimal surgical approach, particularly for prostates ranging from 30 to 80 mL in volume. Notwithstanding technological developments in recent decades that have reduced perioperative morbidity, TURP is associated with complications such as bleeding, transurethral resection syndrome, urethral stricture, and electrolyte imbalance [3]. These constraints have led to the advancement and growing acceptance of alternative methods, such as Holmium Laser Enucleation of the Prostate (HoLEP) [4]. Comparative studies indicate that HoLEP results in shorter hospital stays, reduced blood loss, and shorter catheterization times, despite its extended duration [5,6].

BPH surgery is primarily performed on elderly individuals, who are at an increased likelihood of complications during and postoperatively. A prominent concern within this group is postoperative cognitive dysfunction (POCD), characterized by a decline in cognitive function following surgery. POCD has been associated with increased morbidity, mortality, prolonged hospitalization, and reduced functional independence [7]. Cognitive deterioration following surgery has been extensively studied in cardiac, orthopedic, thoracic, and neurosurgical populations [8]. Nonetheless, data about cognitive outcomes following BPH surgery remain limited and ambiguous.

Previous research investigating cognitive alterations after TURP or other urological procedures has mainly utilized the Mini-Mental State Examination (MMSE). The MMSE is a commonly used assessment with high specificity. However, it is ineffective at detecting mild cognitive impairment, particularly in those with high competence. Consequently, previous studies may have underestimated subtle changes in cognitive performance post-surgery [9,10,11]. Moreover, prior studies have had varying outcomes, and the prevalence, etiology, and significance of cognitive deterioration following BPH surgery remain uncertain [12,13,14].

The Montreal Cognitive Assessment (MoCA) has emerged as a more sensitive screening tool for mild cognitive impairment, particularly in the domains of executive function and attention. The MoCA evaluates multiple cognitive domains, including short-term memory, visuospatial abilities, executive function, attention, working memory, language, and orientation, demonstrating superior sensitivity compared to the MMSE in detecting mild cognitive impairments [12,13].

Given the increasing life expectancy of the surgical population and the potential therapeutic consequences of postoperative cognitive decline, further research is essential. Despite the growing use of perioperative cognitive research, to our knowledge, no study has directly examined the effects of HoLEP and TURP on postoperative cognitive outcomes using MoCA. We hypothesized that, after controlling for age and educational level, the surgical method would not independently influence postoperative cognitive progression at three months; nevertheless, subtle alterations detectable by MoCA may emerge over time.

## 2. Materials and Methods

### 2.1. Study Design and Ethical Approval

This prospective observational cohort study aimed to evaluate the impact of surgical method on postoperative cognitive and psychosocial outcomes in patients receiving TURP or HoLEP for BPH. The Institutional Review Board (IRB) (approval number: 2025/13/1156; date: 1 July 2025, Clinical Trials: NCT07468084) authorized the study protocol, and all procedures adhered to the Declaration of Helsinki. Prior to participation, all individuals were required to provide their written consent. The primary objective of this study was to utilize the MoCA three months post-surgery to examine cognitive alterations following HoLEP and TURP procedures. The secondary objectives were to utilize the MMSE to assess overall cognitive function and to evaluate anxiety and depression levels before, , and following the surgical procedure.

Between 8 July 2025 and 2 December 2025, consecutive patients scheduled for HoLEP or TURP due to symptomatic BPH were assessed for eligibility. To maintain uniformity in surgical indications and procedural intricacy, only patients with a prostate volume between 50 and 100 cc, as measured by transrectal ultrasonography, were included. All patients were consecutively screened in a high-volume tertiary referral center. The choice of surgical modality (HoLEP or TURP) was determined by established institutional protocols and the surgeon’s preference. No allocation was influenced by study-related considerations.

### 2.2. Study Population

Eligible patients were between 55 and 80 years of age and capable of independently completing standardized cognitive and psychological assessments. We included only patients with a baseline MMSE score of 24 or above. This aimed to mitigate the impact of pre-existing neurocognitive impairment and facilitate the observation of post-surgery cognitive function changes. Using MoCA as an exclusion criterion might have reduced the external validity of the study by excluding patients with subclinical cognitive variations. We established this standard to exclude individuals who may have dementia or clinically substantial cognitive impairment at the outset.

The exclusion criteria included confirmed neurodegenerative disorders (e.g., Alzheimer’s disease or Parkinson’s disease), previous cerebrovascular accidents resulting in lasting neurological deficits, traumatic brain injuries, significant psychiatric disorders (such as active major depressive disorder or psychotic disorders), chronic use of centrally acting sedatives or antipsychotic medications, alcohol or substance abuse, severe visual or auditory impairments hindering neurocognitive assessments, prior prostate surgeries, active malignancies. Patients with severe perioperative complications were excluded from the final analysis to minimize confounding effects on postoperative cognitive outcomes.

The conclusive longitudinal analysis comprised solely patients who underwent comprehensive cognitive and psychological evaluations prior to surgery and three months postoperatively*.*

### 2.3. Baseline Preoperative Characteristics

We meticulously documented baseline demographic and clinical parameters to ascertain the comparability of the groups and identify any variables that may have influenced the outcomes. Demographic data included age and years of formal education. The duration of the operation (in minutes) and the volume of the prostate were documented. The clinical status was assessed utilizing the American Society of Anesthesiologists (ASA) physical status categorization and the Charlson Comorbidity Index (CCI), which quantifies the overall burden of comorbidities. Prior to initiating longitudinal modeling, we verified the equivalence of the HoLEP and TURP groups at baseline. This was implemented to mitigate selection bias and enhance internal validity.

### 2.4. Surgical Techniques and Surgeon Experience

The surgical procedure (HoLEP or TURP) was selected according to the patient’s clinical requirements, the prostate’s attributes, and the standard practices of the institution, irrespective of the patient’s participation in the study. To diminish heterogeneity among operators and alleviate any bias linked to procedural learning curves, all procedures were performed by urologists with substantial proficiency in both techniques. Prior to the commencement of the study, each participating surgeon had performed over 50 procedures for each category of operation. This is typically seen as a level of experience sufficient to surpass the learning phase and attain proficiency in the procedure. Both surgical procedures were executed with standardized operating techniques in accordance with institutional protocols [15]. The study aimed to reduce performance bias by standardizing surgical expertise across groups, therefore isolating the independent impact of surgical modality on postoperative cognitive results.

### 2.5. Psychological and Cognitive Assessment

The identical psychometrician administered neurocognitive and psychological assessments at two distinct intervals: prior to the operation (baseline) and three months post-operation.

We documented educational attainment as it is a recognized determinant influencing performance on cognitive assessments and serves as a proxy for cognitive reserve.

A qualified neuropsychologist, unaware of the study hypothesis or surgery group allocation, administered all neurocognitive assessments uniformly.

### 2.6. Anesthetic and Perioperative Standardization

To minimize perioperative confounding variables, our anesthetic and analgesic protocols were strictly standardized. All procedures were performed by the same proficient surgical team under localized (spinal) anesthesia, intentionally avoiding general anesthesia to eliminate its well-documented cognitive risks. Intraoperative hemodynamics were meticulously maintained within established thresholds to prevent prolonged hypotension and hypoxia, both of which can independently impair cognitive function. Furthermore, postoperative analgesia was managed using a standardized non-opioid multimodal approach (e.g., intravenous paracetamol and non-steroidal anti-inflammatory drugs). The use of opioid analgesics was explicitly avoided to prevent pharmacologically induced postoperative delirium.

### 2.7. Statistical Evaluation

#### Sample Size Calculation

We utilized G*Power software (version 3.1.9.7) to determine the sample size in advance. A medium effect size (Cohen’s d = 0.5) was postulated for the primary outcome (alteration in MoCA score at 3 months), grounded in previously published data evaluating postoperative cognitive changes in analogous surgical cohorts. The requisite minimum sample size was established at 66 patients per group, with an alpha level of 0.05 and a statistical power of 80%. Considering the potential for attrition during follow-up, enrollment was subsequently augmented. The final analysis included 132 patients with extensive longitudinal data.

All statistical analyses were conducted using IBM SPSS Statistics. A repeated-measures General Linear Model (GLM) framework was employed to examine the temporal changes in cognitive and psychological outcomes. Time (preoperative vs. postoperative) served as a within-subject factor for each outcome variable (MoCA, MMSE, and BAI), whereas surgical technique (HoLEP vs. TURP) functioned as a between-subject factor.

Age and years of formal education were identified as potential confounding variables and included as continuous covariates in all models. Age is a recognized determinant of postoperative neurocognitive susceptibility, whereas education functions as a measure of cognitive reserve, significantly influencing performance on evaluations such as the MoCA. Adjustments for these variables were thought essential to provide unbiased estimates of the independent effect of surgical mode.

All analyses employed the Type III sum of squares. The estimated marginal means were determined at the average values of the covariates. The Bonferroni adjustment was applied for multiple comparisons. Partial eta squared (η^2^) was utilized to convey effect sizes. The model’s assumptions were rigorously evaluated. Levene’s test assessed homogeneity of variance, Box’s M test evaluated equality of covariance matrices, and Mauchly’s test examined sphericity. The Greenhouse–Geisser adjustment was applied if necessary. Two-tailed statistical tests were employed, with a *p*-value of less than 0.05 considered statistically significant.

The analysis aimed to isolate the independent impact of surgical technique on postoperative cognitive outcomes by incorporating age and educational level as covariates within a longitudinal repeated-measures framework, thereby mitigating bias related to age, educational achievement, and cognitive reserve. Statistical analyses were conducted using SPSS Statistics software, version 25.0 (SPSS Inc., Chicago, IL, USA).

## 3. Results

### 3.1. Patient Flow and Study Cohort

**Figure 1:**
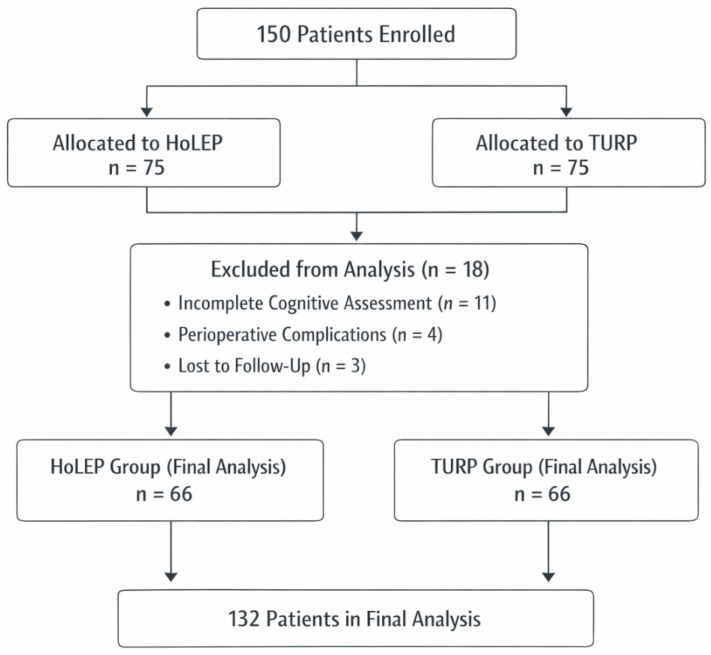
Flowchart for study participants.

One hundred fifty patients registered for the trial in advance. Each surgery cohort (HoLEP and TURP) had 75 patients. In the follow-up, 18 patients were excluded from the final analysis due to incomplete postoperative cognitive assessments (n = 11), perioperative problems requiring additional treatment (n = 4), or loss to follow-up (n = 3). The final study cohort consisted of 132 patients, with 66 in the HoLEP group and 66 in the TURP group, all of whom completed both baseline and 3-month postoperative assessments (Figure 1).

### 3.2. Baseline Characteristics

The two groups had comparable baseline demographic and clinical features (Table 1). No significant differences were seen in age, years of schooling, ASA classification, CCI, or prostate volume (all *p* > 0.05). The groups had comparable cognitive (MoCA and MMSE) and psychological (BAI and BDI) ratings prior to the surgery. This baseline equivalency improves the internal validity of subsequent longitudinal comparisons.

### 3.3. Primary Outcome

#### Montreal Cognitive Assessment (MoCA)

No significant change in preoperative MoCA scores was seen between HoLEP and TURP patients (23.09 ± 1.62 vs. 23.55 ± 1.99, *p* > 0.05). In the modified repeated-measures model, controlling for age and educational attainment, no statistically significant main impact of time was observed (F = 3.661, *p* = 0.058, partial η^2^ = 0.028) (Table 2). A minor decrease in the mean MoCA score was observed three months post-surgery (mean difference = 0.22 points). Nonetheless, this alteration lacks sufficient magnitude to be clinically relevant in cognitive screening instruments. The interaction between time and surgical technique was not significant (F = 0.698, *p* = 0.405, partial η^2^ = 0.005). This indicates that the cognitive trajectory remained consistent over time between HoLEP and TURP. The impact of surgical modality on various patients was not significant (F = 1.974, *p* = 0.162, partial η^2^ = 0.015), indicating that the kind of surgery did not independently predict global cognitive outcomes. Educational attainment was identified as an independent predictor of MoCA performance (F = 5.239, *p* = 0.024, partial η^2^ = 0.039). Conversely, age was not significantly associated with postoperative MoCA changes in the adjusted model. The data indicate that neither HoLEP nor TURP was associated with clinically significant cognitive deterioration at three months post-surgery.

### 3.4. Secondary Outcomes

#### Mini-Mental State Examination

Despite comparable baseline MMSE scores among groups, the adjusted analysis revealed a statistically significant main impact of time (F = 6.081, *p* = 0.015, partial η^2^ = 0.045). Nonetheless, the impact magnitude was minimal, and no significant interaction was observed between time and surgical technique (F = 1.475, *p* = 0.227), indicating that this temporal shift was unrelated to the surgical method employed.

The comparison between groups failed to reach significance. The present prospective cohort study supports the neurocognitive safety of contemporary endoscopic surgical interventions. The results were statistically significant (*p* = 0.056), although they did not conform to the conventional criteria. Age markedly affected time (F = 7.092, *p* = 0.009), signifying heightened heterogeneity in MMSE trajectory among older adults.

## 4. Beck Anxiety Inventory (BAI)

Anxiety scores remained constant over time. No significant main effect of time was observed (*p* = 0.654), no significant interaction effect was detected (*p* = 0.171), and no difference between groups was found (*p* = 0.925). Effect sizes were negligible (partial η^2^ < 0.015), indicating low therapeutic relevance.

## 5. Beck Depression Inventory (BDI)

Throughout the 3-month follow-up period, no significant alterations in depressed symptom scores were observed (*p* = 0.324), and the trajectory did not vary across surgical groups (interaction *p* = 0.278). The intergroup impact was not significant (*p* = 0.539), and the effect sizes were minimal across all analyses (partial η^2^ < 0.01).

## 6. Discussion

The present prospective investigation demonstrated that neither HoLEP nor TURP was associated with notable cognitive decline at three months postoperatively. Upon adjusting for age and educational level, the surgical method did not independently influence cognitive progression as assessed by MoCA. Importantly, there was no notable interaction between time and technique, indicating that postoperative cognitive trajectories were comparable in both groups.

## 7. Postoperative Cognitive Dysfunction in an Expanded Surgical Context

POCD has been observed across multiple surgical disciplines, particularly in the elderly population [7,14]. POCD is commonly reported in major non-urological surgeries, with incidence rates ranging from 10% to 30% after three months in high-risk populations. Evered et al.’s extensive international consensus statement showed that perioperative neurocognitive disorders encompass a spectrum of cognitive alterations influenced by the patient’s susceptibility, rather than solely by the surgical procedure. Although the precise processes remain inadequately elucidated, increasing data indicate perioperative neuroinflammation, systemic stress response, and impairment of the blood–brain barrier [16]. Previous concepts focusing only on hypoxemia have not been reliably corroborated, as cognitive impairment might manifest even without measurable cerebral desaturation [16]. The results demonstrate a complex relationship between perioperative physiologic stresses and pre-existing vulnerabilities.

Most prior studies evaluating cognitive outcomes after BPH surgery relied primarily on the MMSE as the principal screening tool [11,12,17]. The MMSE, while often employed and demonstrating significant specificity, has limited sensitivity in detecting modest executive dysfunction, perhaps resulting in an underassessment of moderate postoperative impairment. Conversely, MoCA evaluates executive and attentional domains more comprehensively and is more effective in detecting moderate cognitive impairment [18]. Given the absence of a substantial time × surgical method interaction in our MoCA-based study, we can be more confident that the type of surgery is unlikely to influence cognitive outcomes in the medium term.

The optimal timing for postoperative cognitive evaluation is still a subject of controversy. Previous investigations have assessed cognitive outcomes from the early postoperative period to one year post-surgery [9,10,11]. A three-month follow-up is usually regarded as a clinically significant period, enabling differentiation between temporary postoperative changes and more persistent neurocognitive modifications [7,14,16].

Hasan et al. emphasized that pre-existing cognitive impairment is a crucial predictor of POCD and highlighted the importance of a systematic preoperative cognitive evaluation [19]. By excluding patients with a baseline MMSE score below 24, our study mitigated the influence of pre-existing cognitive weakness, thereby enhancing internal validity and isolating the surgical effect.

Our findings align with previous research examining cognitive consequences following TURP. Haan et al. conducted a prospective study comparing spinal and general anesthesia in elderly men undergoing TURP [11]. Cognitive performance did not exhibit a significant deterioration at 4 days or 3 months post-surgery [11]. Bennett et al. similarly noted no significant increase in subjective memory complaints or objective cognitive impairment following TURP compared to control surgery patients [12].

These trials indicated minor score variations; nevertheless, they did not demonstrate any long-term deterioration of clinical significance. Our findings support these observations, as the negligible, non-significant decline in MoCA scores did not lead to considerable cognitive impairment. Specifically, the observed mean decrease of approximately 0.22 points in the MoCA score falls well below the widely accepted Minimal Clinically Important Difference (MCID) of 2–3 points for neurocognitive screening, underscoring that this fractional fluctuation lacks clinical relevance.

However, cognitive effects have rarely been directly compared among various methods. Wiedemann et al. evaluated potential cognitive changes post-TURP in comparison to GreenLight laser therapy, suggesting that perioperative factors such as age, comorbidity burden, hemoglobin fluctuations, and electrolyte variations were more critical determinants than the surgical method itself [13]. Consistent with this perspective, our updated analysis identified educational achievement, rather than surgical method, as a significant predictor of cognitive performance. Our findings enhance the current literature by demonstrating that, even when evaluated with MoCA, a more sensitive tool for detecting mild cognitive impairment, cognitive trajectories are similar between HoLEP and TURP. The absence of a substantial interaction effect reinforces the notion that the type of surgery does not exert an independent influence on cognitive results during the study’s midpoint.

### 7.1. Analysis and Clinical Implications

Factors pertaining to the patient exert a greater influence on postoperative cognitive trajectories than those associated with the operation. In our group, educational attainment serving as a surrogate for cognitive reserve demonstrated a substantial association with MoCA performance, thereby supporting the cognitive reserve concept.

These outcomes have tangible implications. In appropriately selected elderly individuals without prior cognitive impairment, modern endoscopic prostate surgery appears to be neurocognitively safe after three months. Thus, the choice between HoLEP and TURP in surgical decision-making may be mostly determined by urological and functional considerations rather than concerns about varying cognitive risks. The present prospective cohort analysis demonstrates that contemporary endoscopic surgical procedures for BPH, whether performed using HoLEP or TURP, do not result in notable cognitive deterioration at three months postoperatively. MoCA, an effective instrument for detecting moderate cognitive impairment, revealed no significant differences in cognitive trajectory between the two approaches after adjusting for age and educational level [18].

Several patients in our cohort had fluctuating cognitive scores; nevertheless, longitudinal modeling did not indicate a sustained decrease associated with the surgical procedure. Longitudinal data reveal that even transient postoperative cognitive changes may be associated with an increased long-term risk of dementia, underscoring the need for continuous monitoring in senior populations.

Educational attainment emerged as an independent predictor of cognitive performance, corroborating the cognitive reserve theory [20]. Stern proposed that individuals with higher educational attainment are more adept at managing neuropathological damage. Our findings align with this concept, as MoCA performance was significantly associated with education but not with surgical technique. Therefore, it was essential to account for educational level to clarify the independent effect of surgical technique.

The psychological measures, including anxiety and depressive symptoms, were stable during follow-up and demonstrated no significant differences between groups. The data indicate that perioperative mood disturbances did not affect cognitive testing. The merits of this study are its prospective design, pre-established adjustments for demographic confounders, standardized perioperative management, and in-person neuropsychological assessments conducted by qualified professionals. Utilizing MoCA as the primary endpoint is a significant methodological advancement over previous BPH research that relied solely on MMSE.

### 7.2. Limitations

Although it possessed sufficient power to detect intermediate effect sizes, it may not have identified minor differences. A notable limitation of our study is that the follow-up period was limited to three months. While three months is a standard milestone to differentiate transient postoperative delirium from persistent neurocognitive disorder, POCD can evolve dynamically. A longer follow-up period (e.g., 6 to 12 months) could potentially reveal delayed-onset cognitive decline or long-term compensatory differences between the two surgical modalities. Future studies with extended follow-up durations and biomarker associations are essential to fully map these long-term neurocognitive trajectories. Another potential limitation is the risk of practice effects resulting from repeated administration of the MoCA and MMSE. However, the three-month interval between tests substantially mitigates this risk. Furthermore, because both surgical groups experienced identical testing schedules, any residual practice effect would be balanced between the cohorts and is unlikely to confound the comparative outcomes. Additionally, this was a single-center study, which may limit the generalizability of the findings to broader populations and different clinical settings. The exclusion of patients with perioperative complications, although intended to reduce confounding, may limit the generalizability of the findings to real-world clinical settings.

## 8. Conclusions

This prospective cohort analysis indicates that contemporary endoscopic surgical treatment of BPH, whether performed via HoLEP or TURP, can be regarded as neurocognitively safe in the mid-term postoperative phase. After adjusting for age and educational attainment, the kind of surgery did not exert an independent influence on cognitive trajectory as assessed by MoCA. In an elderly surgical population where preserving functional independence is essential, our findings provide clinically meaningful confidence for patients and healthcare providers.

## Figures and Tables

**Table 1:** Baseline Demographic and Clinical Characteristics.

Variable	HoLEP (n = 66)	TURP (n = 66)	*p*-Value
Age (years), mean ± SD	68.4 ± 5.2	67.9 ± 5.8	0.62
Education (years), mean ± SD	10.2 ± 3.1	9.8 ± 3.4	0.48
Charlson Comorbidity Index	3.1 ± 1.2	3.0 ± 1.3	0.65
Prostate volume (cc)	85 ± 12	82 ± 15	0.29
Baseline MoCA	23.09 ± 1.62	23.55 ± 1.99	0.37
Baseline MMSE	27.2 ± 1.6	27.0 ± 1.8	0.54
Baseline BAI	7.76 ± 6.75	7.88 ± 6.56	0.73
Baseline BDI	8.44 ± 8.61	7.30 ± 7.56	0.41
Operation Time (min), mean ± SD	68.4 ± 8.3	70.3 ± 7.9	0.1

Data presented as mean ± SD unless otherwise indicated. Independent samples *t*-test, Mann–Whitney U, or chi-square test were used as appropriate.

**Table 2:** Repeated-Measures General Linear Model (GLM) Results Adjusted for Age and Education.

Outcome	Effect	F (df1, df2)	*p*-Value	Partial η^2^
MoCA	Time	F (1, 128) = 3.66	0.058	0.028
	Time × Technique	F (1, 128) = 0.70	0.405	0.005
	Technique	F (1, 128) = 1.97	0.162	0.015
	Education	F (1, 128) = 5.24	0.024	0.039
MMSE	Time	F (1, 128) = 6.08	0.015	0.045
	Time × Technique	F (1, 128) = 1.48	0.227	0.011
	Technique	F (1, 128) = 3.72	0.056	0.028
	Age × Time	F (1, 128) = 7.09	0.009	0.052
BAI	Time	F (1, 128) = 0.20	0.654	0.002
	Time × Technique	F (1, 128) = 1.90	0.171	0.015
	Technique	F (1, 128) = 0.01	0.925	<0.001
BDI	Time	F (1, 128) = 0.98	0.324	0.008
	Time × Technique	F (1, 128) = 1.19	0.278	0.009
	Technique	F (1, 128) = 0.38	0.539	0.003

MoCA: Montreal Cognitive Assessment; MMSE: Mini-Mental State Examination; BAI: Beck Anxiety Inventory; BDI: Beck Depression Inventory. df1 = numerator degrees of freedom; df2 = denominator degrees of freedom. Models adjusted for age and educational level. Partial η^2^ values represent effect size estimates.

## Data Availability

Data are available upon reasonable request.

## Abbreviations

ASA	American Society of Anesthesiologists
BAI	Beck Anxiety Inventory
BDI	Beck Depression Inventory
BOO	Bladder Outlet Obstruction
BPH	Benign Prostatic Hyperplasia
CCI	Charlson Comorbidity Index
GLM	General Linear Model
HoLEP	Holmium Laser Enucleation of the Prostate
IRB	Institutional Review Board
LUTS	Lower Urinary Tract Symptoms
MCID	Minimal Clinically Important Difference
MoCA	Montreal Cognitive Assessment
MMSE	Mini-Mental State Examination
POCD	Postoperative Cognitive Dysfunction
TURP	Transurethral Resection of the Prostate

## References

[1] Wei J.T., Calhoun E., Jacobsen S.J. (2005). Urologic diseases in America project: Benign prostatic hyperplasia. J. Urol..

[2] Patel N.D., Parsons J.K. (2014). Epidemiology and etiology of benign prostatic hyperplasia and bladder outlet obstruction. Indian J. Urol..

[3] Rassweiler J., Teber D., Kuntz R., Hofmann R. (2006). Complications of transurethral resection of the prostate (TURP)—Incidence, management, and prevention. Eur. Urol..

[4] Zarrabi A., Gross A.J. (2011). The evolution of lasers in urology. Ther. Adv. Urol..

[5] Michalak J., Tzou D., Funk J. (2015). HoLEP: The gold standard for the surgical management of BPH in the 21st century. Am. J. Clin. Exp. Urol..

[6] Cornu J.N., Ahyai S., Bachmann A., de la Rosette J., Gilling P., Gratzke C., McVary K., Novara G., Woo H., Madersbacher S. (2015). A systematic review and meta-analysis of functional outcomes and complications following transurethral procedures for lower urinary tract symptoms resulting from benign prostatic obstruction: An update. Eur. Urol..

[7] Monk T.G., Weldon B.C., Garvan C.W., Dede D.E., Van Der Aa M.T., Heilman K.M., Gravenstein J.S. (2008). Predictors of cognitive dysfunction after major noncardiac surgery. Anesthesiology.

[8] Koster S., Hensens A.G., Schuurmans M.J., van der Palen J. (2011). Risk factors of delirium after cardiac surgery: A systematic review. Eur. J. Cardiovasc. Nurs..

[9] Mejia S., Gutierrez L.M., Villa A.R., Ostrosky-Solis F. (2004). Cognition, functional status, education, and the diagnosis of dementia and mild cognitive impairment in Spanish-speaking elderly. Appl. Neuropsychol..

[10] Miyazaki S., Yoshitani K., Miura N., Irie T., Inatomi Y., Ohnishi Y., Kobayashi J. (2011). Risk factors of stroke and delirium after off-pump coronary artery bypass surgery. Interact. Cardiovasc. Thorac. Surg..

[11] Haan J., van Klee J.W., Bloem B.R., Zwartendijk J., Lanser J.B., Brand R., van der Does I.G., Krul E.J., Elshove H.M., Moll A.C. (1991). Cognitive function after spinal or general anesthesia for transurethral prostatectomy in elderly men. J. Am. Geriatr. Soc..

[12] Bennett H.E., Bishop M., Zadik T.D., Lincoln N.B. (2004). Cognitive impairment after transurethral resection of the prostate (TURP). Disabil. Rehabil..

[13] Wiedemann A., Maykan R., Pennekamp J., Hirsch J., Heppner H. (2015). Potential cognitive alterations after treatment of benign prostate syndrome: Investigations on transurethral electroresection and 180 W GreenLight XPS laser therapy. Z. Gerontol. Geriatr..

[14] The Nomenclature Consensus Working Group (2018). Recommendations for the nomenclature of cognitive change associated with anaesthesia and surgery—2018. Br. J. Anaesth..

[15] Shah H.N., Mahajan A.P., Sodha H.S., Hegde S., Mohile P.D., Bansal M.B. (2007). Prospective evaluation of the learning curve for holmium laser enucleation of the prostate. J. Urol..

[16] Rasmussen L.S. (2006). Postoperative cognitive dysfunction: Incidence and prevention. Lancet.

[17] Chi Y.L., Li Z.S., Lin C.S., Wang Q., Zhou Y.K. (2017). Evaluation of the postoperative cognitive dysfunction in elderly patients with general anesthesia. Eur. Rev. Med. Pharmacol. Sci..

[18] Nasreddine Z.S., Phillips N.A., Bédirian V., Charbonneau S., Whitehead V., Collin I., Cummings J.L., Chertkow H. (2005). The Montreal Cognitive Assessment (MoCA): A brief screening tool for mild cognitive impairment. J. Am. Geriatr. Soc..

[19] Hasan T.F., Kelley R.E., Cornett E.M., Urman R.D., Kaye A.D. (2020). Cognitive impairment assessment and interventions to optimize surgical patient outcomes. Best. Pract. Res. Clin. Anaesthesiol..

[20] Stern Y. (2012). Cognitive reserve in ageing and Alzheimer’s disease. Lancet Neurol..

